# Hybrid Proteins with Short Conformational Epitopes of the Receptor-Binding Domain of SARS-CoV-2 Spike Protein Promote Production of Virus-Neutralizing Antibodies When Used for Immunization

**DOI:** 10.1134/S0006297922040022

**Published:** 2022-04-08

**Authors:** Anna S. Karyagina, Alexander V. Gromov, Tatyana M. Grunina, Alexander M. Lyaschuk, Maria S. Poponova, Denis A. Kleymenov, Natalia V. Strukova, Maria S. Generalova, Anna V. Ryazanova, Zoya M. Galushkina, Olga Yu. Dobrynina, Tatyana N. Bolshakova, Maria V. Sergeeva, Ekaterina A. Romanovskaya-Romanko, Igor V. Krasilnikov, Marina E. Subbotina, Vladimir G. Lunin

**Affiliations:** 1 Gamaleya National Research Center of Epidemiology and Microbiology, Ministry of Health of the Russian Federation, 123098 Moscow, Russia; 2 All-Russia Research Institute of Agricultural Biotechnology, 127550 Moscow, Russia; 3 Belozersky Institute of Physico-Chemical Biology, Lomonosov Moscow State University, 119992 Moscow, Russia; 4 Institute of Influenza, Ministry of Health of the Russian Federation, 197376 St. Petersburg, Russia; 5 Saint Petersburg Institute of Vaccines and Sera, FMBA, 198320 St. Petersburg, Russia

**Keywords:** SARS-CoV-2, S protein, RBD, RBM, epitope vaccine, epitope, aldolase

## Abstract

Based on the previously developed approach, hybrid recombinant proteins containing short conformational epitopes (a.a. 144-153, 337-346, 414-425, 496-507) of the receptor-binding domain (RBD) of SARS-CoV-2 Spike protein (S protein) were synthesized in *Escherichia coli* cells as potential components of epitope vaccines. Selected epitopes are involved in protein–protein interactions in the S protein complexes with neutralizing antibodies and ACE2 (angiotensin-converting enzyme 2). The recombinant proteins were used for immunization of mice (three doses with 2-week intervals), and the immunogenicity of protein antigens and ability of the resulting sera to interact with inactivated SARS-CoV-2 and RBD produced in eukaryotic cells were examined. All recombinant proteins showed high immunogenicity; the highest titer in the RBD binding assay was demonstrated by the serum obtained after immunization with the protein containing epitope 414-425. At the same time, the titers of sera obtained against other proteins in the RBD and inactivated virus binding assays were significantly lower than the titers of sera obtained with the previously produced four proteins containing the loop-like epitopes 452-494 and 470-491, the conformation of which was fixed with a disulfide bond. We also studied activation of cell-mediated immunity by the recombinant proteins that was monitored as changes in the levels of cytokines in the splenocytes of immunized mice. The most pronounced increase in the cytokine synthesis was observed in response to the proteins containing epitopes with disulfide bonds (452-494, 470-491), as well as epitopes 414-425 and 496-507. For some recombinant proteins with short conformational epitopes, adjuvant optimization allowed to obtained mouse sera displaying virus-neutralizing activity in the microneutralization assay with live SARS-CoV-2 (hCoV-19/Russia/StPetersburg-3524/2020 EPI_ISL_415710 GISAID). The results obtained can be used to develop epitope vaccines for prevention of COVID-19 and other viral infections.

## INTRODUCTION

Development of new vaccines for prevention of COronaVIrus Disease 2019 (COVID-19) and other viral infections is a very important task. Whole-virus vaccines, protein subunit and full-size protein vaccines, vector-based vaccines, and RNA vaccines (which ensure the synthesis of viral proteins in an organism) induce generation of a wide array of antibodies, although not all of these antibodies are protective [1]. In some cases, such antibodies can promote antibody-dependent enhancement of infection when the disease is contracted after vaccination [2-4]. Vaccines containing small epitopes of infectious agent proteins can avoid the abovementioned problems. They are also easily standardized and exhibit low reactogenicity. The drawbacks of epitope vaccines are low immunogenicity and inability to induce formation of neutralizing antibodies, as well as possible capacity of new virus variants to avoid immune response developed to these vaccines. Some of the approaches used to increase vaccine immunogenicity and to facilitate the synthesis of virus-neutralizing antibodies involve epitope multimerization [5] and adjuvant optimization [6]. Another promising approach is selection of continuous conformational epitopes (elements of protein surface capable of effective induction of antibody synthesis) instead of linear epitopes, since the structure of the former is more similar to the structure of viral protein. Based on the contacts identified in the S protein complexes with neutralizing antibodies and ACE2 (angiotensin-converting enzyme 2), we selected two determinants in the S protein receptor-binding motif (RBM) (a.a. 452-494 and 470-491) that participated in a large number of contacts between the S protein and neutralizing antibodies or ACE2 [7]. Both determinants have a loop-like conformation and include two cysteine residues that form a disulfide bond in the S protein. For immunization, we produced two hybrid proteins, in which the selected antigenic determinants were inserted into the turn of the helix-turn-helix motif of *Methylococcus capsulatus* Rop-like protein in order to bring together the N- and C-termini and to preserve the loop-like conformation of the epitopes (i.e., the Rop-like protein served as an epitope scaffold). The synthesis of these proteins in *Escherichia coli* cells and the following purification resulted in the disulfide bond formation in the protein structure [7]. The hybrid proteins also contained the heparin-binding domain (HBD) to facilitate protein purification and either *Thermotoga maritima* aldolase (ALD) or α-helical fragment (a.a. 958-991) of the SARS-CoV-2 S protein to ensure protein trimerization. High immunogenicity and ability of antibodies formed in response to immunization with these proteins to interact with the S protein receptor-binding domain (RBD) and inactivated SARS-CoV-2 were demonstrated for all four hybrid proteins.

The objectives of this study were production of other hybrid proteins carrying conformational epitopes of the SARS-CoV-2 S protein according to the previously developed protocol, investigation of immunogenicity of the produced proteins and interaction of induced antibodies with the RBD and inactivated SARS-CoV-2, characterization of cytokine response in splenocytes after immunization with the synthesized proteins, investigation of virus neutralization by the sera obtained with most promising hybrid proteins in the microneutralization assay with the clinical isolate of SARS-CoV-2.

## MATERIALS AND METHODS

**Strain and vectors.** *E. coli* BL21 (DE3) (*E. coli* B F^–^ *dcm* *ompT* *hsdS*(r_B_^–^m_B_^–^) *gal* λ(DE3) cells (Agilent Technologies, USA), modified plasmid vector pQE6 (Qiagen, USA) containing T7 promoter instead of T5 promoter, and pRep4 plasmid from *E. coli* M15 [pRep4] (Qiagen) were used in the study.

**Design of constructs encoding Rop-D2-Rop-Tri-HBD, Rop-D3-Rop-Tri-HBD, Rop-D2-Rop-ALD-HBD, and Rop-D3-Rop-ALD-HBD** **proteins** was described in detail in [7]. These proteins contained partial D2 and D3 sequences (a.a. 470-490 and 453-494, respectively) of the SARS-CoV-2 S protein (UniProtKB: locus SPIKE_SARS2, accession P0DTC2) inserted between two α-helices of the Rop-like protein from *M. capsulatus* (first Rop helix: a.a. 2-34; second Rop helix: a.a. 34-65; PDB: 2JS5_A, RefSeq WP_010959602). Next, sequences coding for either trimerization-mediating α-helix of S protein (Tri) (a.a. 958-991, UniProtKB: locus SPIKE_SARS2, accession P0DTC2) or *T. maritima* ALD (a.a. 2-201, PDB: 1WA3) were introduced. All recombinant proteins contained the HBD (a.a. 160-174, UniProtKB/Swiss-Prot: A1KFU9.1) of the heparin-binding hemagglutinin (HBHA) from *Mycobacterium tuberculosis* at the C-terminus. Sequences coding for linkers and restriction nuclease sites were added to the constructs.

**Rop-D4-Rop-Tri-HBD, Rop-D4-Rop-ALD-HBD, Rop-D6-Rop-Tri-HBD, Rop-D6-Rop-ALD-HBD, Rop-D8-Rop-Tri-HBD, Rop-D8-Rop-ALD-HBD, Rop-D13-Rop-Tri-HBD, Rop-D13-Rop-ALD-HBD, and Rop-RBM-Rop-Tri-HBD** **proteins** were produced as described by Karyagina et al. [7]. The sequences for the S protein fragments D4 (a.a. 496-507), D6 (a.a. 144-153), D8 (a.a. 337-346), D13 (a.a. 414-425), and RBM (a.a. 433-511) were synthesized after codon optimization with the JCat program (http://www.jcat.de/) and correction with the purpose of improving the secondary structure of the transcribed RNAs for further facilitation of their expression in *E. coli* cells (DINAMelt web server; http://www.unafold.org/Dinamelt/applications/two-state-melting-folding.php). All sequences were synthesized by Evrogen (Russia) and cloned into the pL1003 plasmid encoding the Rop-D2-Rop-Tri-HBD protein and pL989 plasmid encoding the Rop-D2-Rop-ALD-HBD protein [7] by substituting the D2 fragment.

**Cultivation of producer strains and disruption of** ***E. coli*** **cells** were carried out as described by Karyagina et al. [7]. Rop-D4-Rop-Tri-HBD, Rop-D8-Rop-Tri-HBD, Rop-D13-Rop-Tri-HBD, and Rop-RBM-Rop-Tri-HBD proteins were synthesized as insoluble inclusion bodies, while Rop-D13-Rop-ALD-HBD, Rop-D4-Rop-ALD-HBD, and Rop-D8-Rop-ALD-HBD were produced in a soluble form. No synthesis of Rop-D6-Rop-ALD-HBD was observed.

**Protein purification** of soluble and insoluble proteins was carried according to the previously published protocols [7]. Proteins synthesized as inclusion bodies were solubilized in 8 M urea in 10 mM Tris-HCl (pH 8.0) and purified on a WorkBeads 40S column (Bio-Works, Sweden) equilibrated with 8 M urea in 10 mM Tris-HCl (pH 8.0). The proteins were eluted with a linear gradient of NaCl (0-1 M) in the same buffer followed by dialysis against 4 M urea in 25 mM Tris-HCl (pH 8.0) for 24 h at 4°C and centrifugation for 30 min at 9000*g*. Next, the proteins were subjected to affinity chromatography on heparin-Sepharose CL-6B (GE Healthcare, USA) equilibrated with 4 M urea in 20 mM Tris-HCl (pH 8.0) and eluted with a linear gradient of NaCl (0-1 M) in 4 M urea, 25 mM Tris-HCl (pH 8.0) followed by dialysis against 25 mM Tris-HCl (pH 6.8) with a stepwise decrease in the urea concentration (to 0 M urea, 0.5 M steps) for 24 h at 4°C.

Soluble proteins were purified by chromatography on a WorkBeads 40S column equilibrated with 20 mM Tris-HCl (pH 8.0). The proteins were eluted with a linear gradient of NaCl (0-1 M) in the same buffer followed by dialysis against 20 mM Tris-HCl (pH 8.0) for 24 h and centrifugation for 30 min at 9000*g*. Next, affinity chromatography was performed on heparin-Sepharose equilibrated with 20 mM Tris-HCl (pH 8.0). The proteins were eluted with 0-1 M gradient of NaCl in 20 mM Tris-HCl (pH 8.0). Obtained protein fractions were dialyzed against 20 mM Tris-HCl (pH 8.0), 50 mM NaCl for 24 h at 4°C with three buffer changes.

The concentration and total amount of protein were determined from the absorbance of protein solutions at 280 nm using bicinchoninic acid. Proteins’ preparations were lyophilized and used in further studies.

The molecular mass and theoretical values of some parameters of the recombinant proteins were calculated using the Expasy on-line service (https://web.expasy.org/protparam/).

**Immunogenic compositions and immunization experiments.** Obtained recombinant proteins (including four previously produced proteins) containing conformational epitopes of SARS-CoV-2 S protein were used for preparing immunogenic compositions containing 1 nmol of the recombinant protein and respective adjuvant in a single dose (150 µl). In the first experiment, mice were immunized with seven recombinant proteins (proteins 5-11 in Table 1) mixed with adjuvant 1 containing 1 mg of diethylaminoethyl (DEAE) dextran 500, 75 µl (50%) of montanide ISA 201 (Seppic SA, France), and 1.5 µl (1%) of retinol palmitate (100,000 IU/ml; Retinoidy, Russia). The sera produced by immunization were evaluated for the antigen immunogenicity and ability to interact with RBD and inactivated SARS-CoV-2 in the first microneutralization assay and then in splenocytes (cell-mediated immunity). The same recombinant proteins were used in the second immunization experiment together with Rop-RBM-Rop-Tri-HBD, RBD (Mount Sinai, USA), and previously produced recombinant proteins (proteins 1-4 in Table 1) [7]. Adjuvant 2 added to the immunogenic compositions contained 1 mg of DEAE-dextran 500, 0.1 µg of lipopolysaccharide (LPS) of *E. coli* O55:B5 (Sigma, USA), and 30 µg of CpG oligodeoxynucleotides (ODN 1826) (Evrogen). The produced sera were assayed in the second microneutralization reaction.

**Table 1:** Characteristics of produced recombinant proteins

No.	Protein	Molecular mass, Da	Protein location after biomass disruption	Theoretical isoelectric point (p*I*)	Absorbance of protein aqueous solution (1 mg/ml) at 280 nm (fully oxidized form)	Absorbance of protein aqueous solution (1 mg/ml) at 280 nm (reduced form)
1	Rop-D2-Rop-Tri-HBD	18,714	inclusion bodies	8.83	0.325	0.318
2	Rop-D3-Rop-Tri-HBD	21,359	inclusion bodies	9.39	0.355	0.349
3	Rop-D2-Rop-ALD-HBD	36,102	soluble fraction	8.58	0.486	0.483
4	Rop-D3-Rop-ALD-HBD	38,747	soluble fraction	9.1	0.491	0.488
5	Rop-D4-Rop-Tri-HBD	17,491	inclusion bodies	9.45	0.256	–
6	Rop-D8-Rop-Tri-HBD	17,627	inclusion bodies	9.49	0.169	–
7	Rop-D4-Rop-ALD-HBD	34,878	soluble fraction	9.06	0.457	–
8	Rop-D8-Rop-ALD-HBD	35,014	soluble fraction	9.07	0.412	–
9	Rop-D6-Rop-Tri-HBD	34,966	inclusion bodies	9.05	0.456	–
10	Rop-D13-Rop-ALD-HBD	35,215	soluble fraction	9.13	0.495	–
11	Rop-D13-Rop-Tri-HBD	17,827	inclusion bodies	9.29	0.334	–
12	Rop-RBM-Rop-Tri-HBD	245,628	inclusion bodies	8.43	0.836	0.831

Note. No absorbance of protein aqueous solution at 280 nm was studied for the reduced proteins with no disulfide bond. The data for proteins 1-4 were obtained previously [7] and are presented for comparison.

Detailed immunization protocols were described in [7]. Female mice (body weight, 18-20 g) of the syngeneic inbred line BALB/c (Gamaleya National Research Center of Epidemiology and Microbiology, Ministry of Health of the Russian Federation) were used for immunization at the age of 5-6 weeks. Mice of the experimental group (*n* = 7) were injected three times every 2 weeks with 150 µl of immunogenic compositions containing recombinant proteins. Animals of the control group (*n* = 7) were injected with 150 µl of physiological saline. Two weeks after the last injection, mice from the experimental and control groups were anaesthetized by isoflurane inhalation (Piramal Enterprises, India) and their blood was collected from the heart. In the first immunization experiment, we also isolated the spleen in order to evaluate activation of the cell-mediated immunity (see below). Vacuum tubes with blood samples were incubated at room temperature for 20 min and centrifuged for 30 min at 1500*g* without refrigeration. The supernatant (blood serum) was transferred into 1.5-ml sterile tubes and kept frozen at –80°C.

**Enzyme-linked immunoassay (ELISA).** Proteins used for immunization, formaldehyde-inactivated SARS-CoV-2, and RBD were adsorbed on the microplates and used as antigens in ELISA. Preparation of inactivated SARS-CoV-2 and RBD, as well as detailed ELISA protocol, were described in [7]. Briefly, the antigens were adsorbed in 96-well Maxisorp microplates (Thermo Scientific, Denmark) overnight at 4°C. After washing, the plates were incubated for 2 h with blocking buffer (PBS containing 5% fat-free milk; TF Ditol, Russia), washed, and incubated with 10-fold serial dilutions of mouse sera. Goat anti-mouse IgG (H+L) conjugated with horseradish peroxidase (Invitrogen, USA) were used as secondary antibodies at 1 : 1200 dilution (100 µl per well). TMB solution (Bioservis, Russia) was used as an HRP substrate for colorimetric detection; the reaction was stopped by adding 1 N H_2_SO_4_. Absorbance at 450 nm was measured with a plate reader with LVF monochromators CLARIOstar (BMG Labtech, USA).

**Activation of cell-mediated immunity** was evaluated based on the level of cytokine synthesis by splenocytes from immunized mice using the multiplex immunological assay with the MILLIPLEX MAP Mouse Cytokine/Chemokine Magnetic Bead Panel (Merck-Millipore, USA) that allows to measure the levels of 32 regulatory, pro- and anti-inflammatory cytokines, chemokines, and growth factors [G-CSF, GM-CSF, IFN-γ, IL-1α, IL-1β, IL-2, IL-3, IL-4, IL-5, IL-6, IL-7, IL-9, IL-10, IL-12 (p40), IL-12 (p70), IL-13, IL-15, IL-17, IP-10, KC, LIF, LIX, MCP-1, M-CSF, MIG, MIP-1α, MIP-1β, MIP-2, RANTES, TNF-α, VEGF, eotaxin/CCL11]. All assays were conducted in accordance with manufacturer’s protocol using recommended sample dilutions and concentrations for the standard curve. The measurements were carried out with a MAGPIX instrument equipped with an xPONENT version 4.2 software (Luminex Corp., USA).

Splenocytes (macrophages, dendritic cells, T and B lymphocytes) were isolated from the mouse spleen 2 weeks after the first immunization experiment. Euthanized mice were washed with 96% alcohol; their abdominal cavity was opened under aseptic conditions; the spleen was isolated and placed into sterile cold DMEM medium (PanEko, Russia). Splenocytes were isolated using a Tissue Grinder Homogenizer Kit (Sigma). All instruments were sterilized by washing in 96% alcohol followed by flaming. An instrument for tissue disruption was assembled under sterile conditions; 100- and 200-mesh cell sieves (Sigma) were used. The spleen was cut into pieces and passed through the sieve into 9-cm Petri dishes with ice-cold DMEM. The resulting cell suspension was transferred into 15-ml sterile centrifuge tubes and centrifuged at 200*g* for 15 min. Pelleted cells were re-suspended in the erythrocyte lysis buffer (150 mM NH_4_Cl, 10 mM KHCO_3_, 1 mM EDTA, pH 7.0-7.6) and incubated for 40 s in the dark on ice followed by dilution with ice-cold DMEM and centrifugation for 15 min at 200*g*. The supernatant was discarded and white cell precipitate was resuspended in complete RPMI-1640 medium without glutamine (PanEko) supplemented with 10% FBS (HyClone, USA), 2 mM L-glutamine (PanEko), antibiotic/antimycotic (Sigma), and 0.05 mM 2-mercaptoethanol (Sigma). The cells in the suspension were counted in a Goryaev chamber. The concentration of cell suspension was adjusted to 5 × 10^6^-10^7^ cell/ml and aliquots (100 µl) were placed into the microplate wells. The cells were incubated for 1-1.5 h in a CO_2_ incubator, after which dilutions of antigens and controls were introduced into each well. The microplate was incubated for 18-24 h in a CO_2_ incubator, after which the culture fluid was transferred into microtubes, frozen, and stored at –80°C prior to analysis.

Splenocytes produced in the first immunization experiment (using proteins 5-11 in Table 1 with adjuvant 1) were analyzed simultaneously with splenocytes isolated after immunization with proteins 1-4 from Table 1 with adjuvant 1 [7] (the plates were stored at –80°C prior to analysis).

**Microneutralization assay.** Neutralizing antibodies were detected using microneutralization assay based on the Median Tissue Culture Infectious Dose (TCID50) [8]. Mouse sera obtained after three immunization rounds with 2-week intervals were heated for 60 min at 56°C to avoid the complement-mediated reduction in the viral activity. Next, a series of 2-fold dilutions of the sera with MEM supplemented with 2% HI-FBS was prepared, starting with 1 : 10 dilution of the whole serum. Each dilution (60 µl) was mixed with an equal volume of the suspension of live SARS-CoV-2 (hCoV-19/Russia/StPetersburg-3524/2020 EPI_ISL_415710 GISAID) containing 25 TCID50/50 µl and incubated for 1 h at 37°C. Next, 100 µl of dilutions were transferred into 96-well plates with a monolayer of Vero cells (ATCC CCL-81). The plates were incubated at 37°C in 5% CO_2_ for 4 days and then tested for the cytopathic effect (CPE). Neutralization was registered when 100% cells in the well were preserved, without visible plaques or CPE. The neutralization titer was calculated as a value reciprocal to the highest dilution for which the neutralizing effect was observed [9].

Experiments on microneutralization were performed in two rounds. In the first microneutralization reaction, we tested the sera obtained in this work and in previous studies [7] after immunization with Rop-D2-Rop-Tri-HBD, Rop-D3-Rop-Tri-HBD, Rop-D2-Rop-ALD-HBD, Rop-D3-Rop-ALD-HBD, Rop-D4-Rop-Tri-HBD, Rop-D4-Rop-ALD-HBD, and Rop-D13-Rop-Tri-HBD with adjuvant 1. In the second experiment, the sera obtained after immunization with the same proteins and with Rop-RBM-Rop-Tri-HBD and RBD (Mount Sinai) with adjuvant 2 were tested. All experiments were conducted at a Biosafety Level 3 (BSL3) laboratory.

**Statistical analysis** was performed using Statistica 12.0 package (Statsoft, USA). The normality of distribution was estimated with the Kolmogorov–Smirnov test. The data are presented as mean ± standard deviation (SD) or as geometric mean ± standard geometric deviation of logarithm base 2 of the antibody titer in the serum. Statistical significance of differences was evaluated using one-way ANOVA with the *post hoc* Tukey’s test. The differences were considered significant at *p* < 0.05.

## RESULTS

**Fig. 1.**
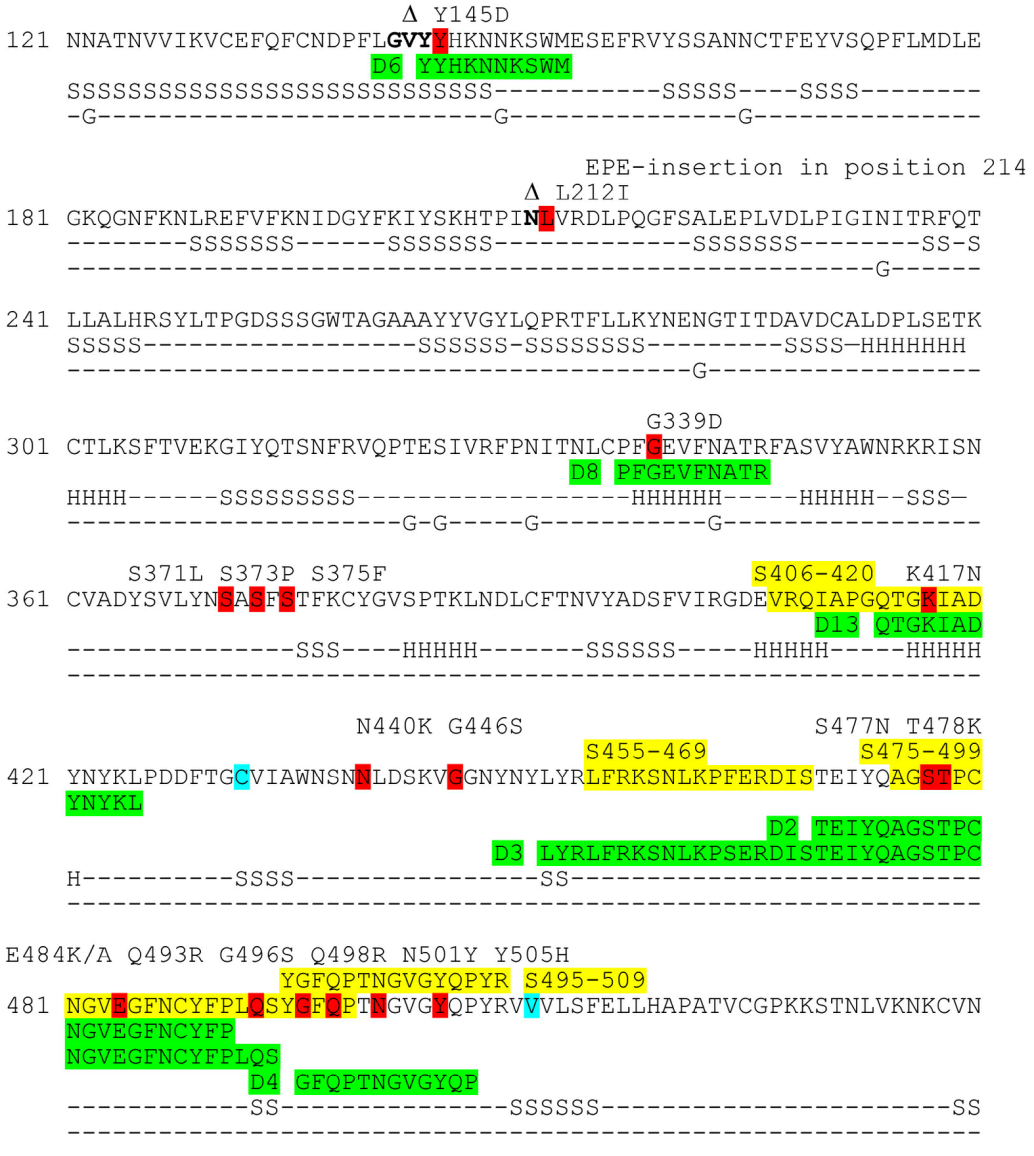
Fragments of the S protein sequence and epitopes used in the study (highlighted with green). Amino acids included in the linear epitopes S406-420, S455-469, S475-499, and S495-509 (data from Lu et al. [5]) that overlap with the sequences of investigated conformational epitopes are highlighted with yellow. Amino acid residues varying in different SARS-CoV-2 variants are shown in red. Amino acid residues lacking in the S protein of the Omicron variant (B.1.1.529) are shown in semi-bold font and indicated with symbol Δ. Amino acid residues at the start and the end of the S protein fragment in the Rop-RBM-Rop-Tri-HBD construct are shown in cyan. Secondary structure elements (α-helices (H), β-strands (S)), and glycosylation sites (G) are marked below the amino acid sequence in accordance with the structural data on the S protein complex with the neutralizing antibody (chain A, PDB: 7C2L).

**Design of recombinant proteins.** Based on the structure of SARS-CoV-2 S protein in complexes with ACE2 and neutralizing antibodies, four surface epitopes were identified – D4 (a.a. 496-507), D6 (a.a. 144-153), D8 (337-346 a.a.), and D13 (414-425 a.a.) (Fig. 1) – in addition to the two previously identified epitopes D2 (a.a. 470-490) and D3 (a.a. 453-494) [7]. All determinants (except D8 with an α-helical conformation) had a loop-like structure with the N- and C-termini brought close together. Recombinant proteins with the D4, D6, D8, and D13 epitopes were organized similarly to the previously obtained proteins with the D2 and D3 epitopes [7]. All epitopes were flanked with α-helices of the Rop-like protein from *M. capsulatus* for additional fixation of N- and C-termini in order to provide the conformational stability of the epitopes. The loop-like determinants were inserted directly into the turn between the helices. In the case of D8 epitope, flexible glycine-containing spacers were introduced between the epitope and α-helices of the Rop-like proteins. To ensure multimerization (in this case, trimerization) of epitopes, we used the α-helix participating in S protein trimerization (Tri) or ALD from *T. maritima* (ALD). The recombinant proteins also contained the HBD of HBHA from *M. tuberculosis*.

Molecular masses of monomeric proteins were in the range of 17-19 kDa (proteins with the Tri sequence) and 35-39 kDa (proteins with the ALD sequence), i.e., were sufficient for the induction of antibody production if trimerization takes place.

The Rop-RBM-Rop-Tri-HBD hybrid protein was designed as a control to investigate the virus-neutralizing activity of the sera. It contained a fragment of the S protein sequence (a.a. 433-511) including D2, D3, and D4 determinants introduced into the turn between the two helices of the Rop-like protein.

**Synthesis and purification of recombinant proteins.** The genes for the recombinant proteins were cloned into the previously generated constructs [7] as described in “Materials and Methods” section. All recombinant proteins were highly expressed except for Rop-D6-Rop-ALD-HBD, which was omitted from the study. Proteins containing S protein trimerization domain (Tri) were produced as inclusion bodies, while ALD-containing proteins were expressed in a soluble form. Therefore, protein purification was conducted under denaturing conditions in the presence of urea (Tri-containing proteins) or under non-denaturing conditions (proteins with ALD). Chromatography on WorkBeads 40S was used as the first purification step for all proteins; the second purification step was affinity chromatography on heparin-Sepharose (because of the presence of HBD in protein structure).

Molecular masses of recombinant proteins calculated based on the amino acid sequence and theoretical values of some protein parameters are presented in Table 1 (the data for the previously produced proteins with epitopes D2 and D3 [7] are included in Table 1 for comparison).

**Fig. 2.**
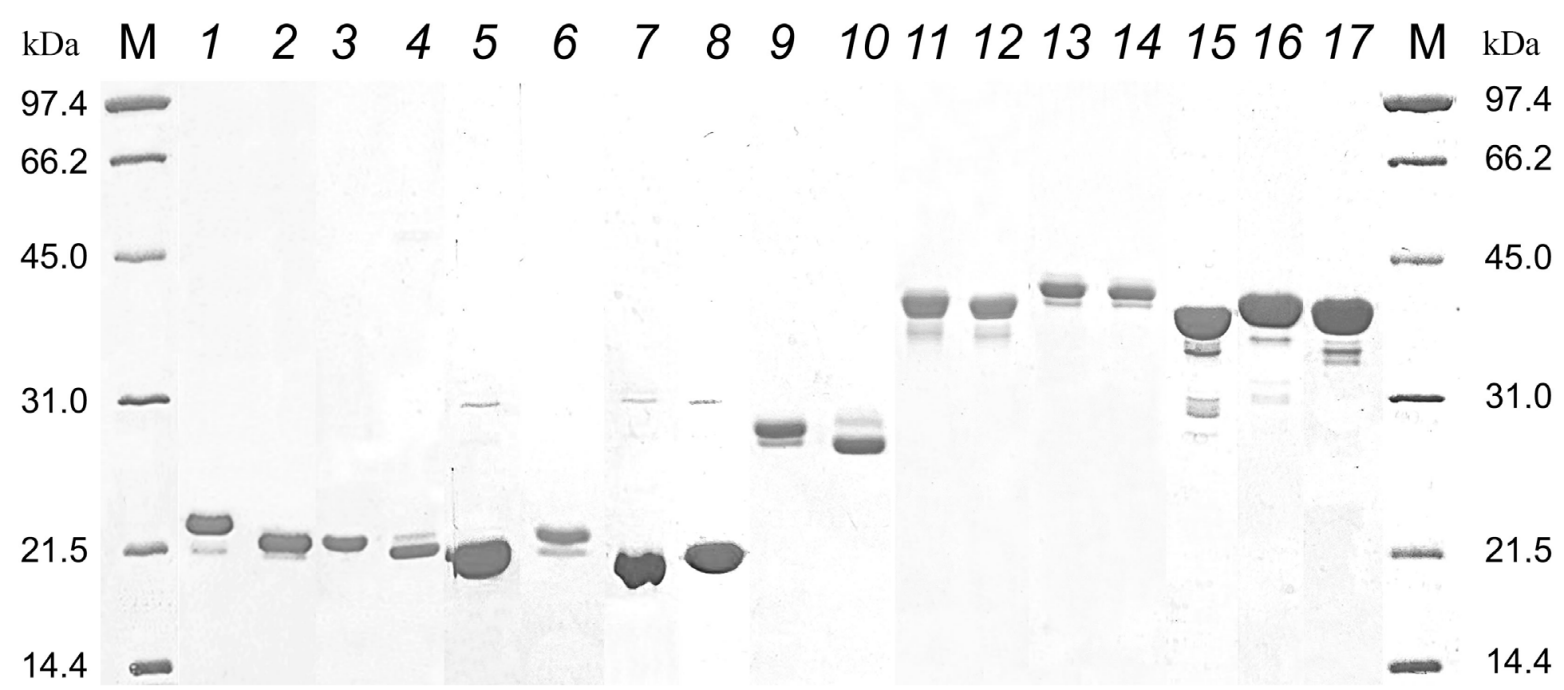
Electrophoresis of recombinant proteins in 12% PAAG. Lanes: M, molecular mass markers, 14.4-97.4 kDa (Bio-Rad, USA); *1*) Rop-D2-Rop-Tri-HBD + DTT; *2*) Rop-D2-Rop-Tri-HBD, no DTT; *3*) Rop-D3-Rop-Tri-HBD + DTT; *4*) Rop-D3-Rop-Tri-HBD, no DTT; *5*) Rop-D4-Rop-Tri-HBD; *6*) Rop-D8-Rop-Tri-HBD; *7*) Rop-D6-Rop-Tri-HBD; *8*) Rop-D13-Rop-Tri-HBD; *9*) Rop-RBM-Rop-Tri-HBD + DTT; *10*) Rop-RBM-Rop-Tri-HBD, no DTT; *11*) Rop-D2-Rop-ALD-HBD + DTT; *12*) Rop-D2-Rop-ALD-HBD, no DTT; *13*) Rop-D3-Rop-ALD-HBD + DTT; *14*) Rop-D3-Rop-ALD-HBD, no DTT; *15*) Rop-D4-Rop-ALD-HBD; *16*) Rop-D8-Rop-ALD-HBD; *17*) Rop-D13-Rop-ALD-HBD.

The results of electrophoretic separation of purified proteins are shown in Fig. 2. Different electrophoretic mobility in the presence and absence of reducing agent dithiothreitol (DTT) of proteins with the D2 and D3 epitopes containing two cysteine residues indicates that the purified proteins formed disulfide bonds. The presence of disulfide bond in the proteins with the D2/D3 determinants was previously demonstrated by mass-spectrometry analysis of tryptic peptides [7].

**Immunogenic compositions and immunization of mice.** Antigenic compositions used for immunization of BALB/C mice were prepared using seven recombinant proteins with the loop-like epitopes (proteins 5-11 in Table 1) and adjuvant 1. The presence of specific class G immunoglobulins (IgGs) in the mouse sera was determined 2 weeks after three immunizations with 2-week intervals using these proteins, as well as formaldehyde-inactivated SARS-CoV-2 and RBD, as antigens. Experimental results and the data obtained earlier for the proteins containing D2 and D3 epitopes are presented in Table 2. All hybrid proteins induced formation of high-titer IgGs that interacted with the antigens used for immunization. It was shown earlier [7] that proteins containing D2 and D3 determinants produced high-titer antibodies that also interacted with the inactivated virus and RBD. The serum against Rop-D13-Rop-Tri-HBD also interacted with the RBD. All proteins with the ALD domain, Rop-D13-Rop-Tri-HBD, and Rop-D4-Rop-Tri-HBD were found to be highly immunogenic. The most pronounced response to the RBD was observed with the serum against the Rop-D3-Rop-ALD-HBD protein. The most pronounced response to inactivated SARS-CoV-2 virus was observed for the animals immunized with Rop-D2-Rop-ALD-HBD.

**Table 2:** Titers of antibodies against different antigens in the mouse sera after three immunizations as determined by ELISA and in virus neutralization experiments

Mouse group	Protein	Antigen used for immunization	Formaldehyde-inactivated SARS-CoV-2	RBD	GMT in microneutralization reaction (range)
1	Physiological saline	< 7.0 ± 1.00	< 7.0 ± 1.00	< 7.0 ± 1.00	5.0
2	Rop-D2-Rop-Tri-HBD	14.21 ± 1.04	8.64 ± 1.00	10.73 ± 1.12	7.4 (5; 10)
3	Rop-D3-Rop-Tri-HBD	13.48 ± 1.19	9.67 ± 1.18	11.44 ± 1.19	5.0
4	Rop-D4-Rop-Tri-HBD	> 15.64 ± 1.00	< 7.0 ± 1.00	< 7.0 ± 1.00	8.2 (5; 20)
5	Rop-D6-Rop-Tri-HBD	10.57 ± 1.40	< 7.0 ± 1.00	< 7.0 ± 1.00	–
6	Rop-D8-Rop-Tri-HBD	8.64 ± 1.32	< 7.0 ± 1.00	< 7.0 ± 1.00	–
7	Rop-D13-Rop-Tri-HBD	> 15.64 ± 1.00	< 7.0 ± 1.00	10.12 ± 1.16	6.7 (5; 10)
8	Rop-D2-Rop-ALD-HBD	> 15.64 ± 1.00	9.88 ± 1.12	11.36 ± 1.17	5.5 (5; 10)
9	Rop-D3-Rop-ALD-HBD	> 15.64 ± 1.00	9.75 ± 1.10	12.61 ± 1.08	5.5 (5; 10)
10	Rop-D4-Rop-ALD-HBD	> 15.64 ± 1.00	< 7.0 ± 1.00	< 7.0 ± 1.00	5.5 (5; 10)
11	Rop-D8-Rop-ALD-HBD	> 15.64 ± 1.00	< 7,0 ± 1.00	< 7.0 ± 1.00	–
12	Rop-D13-Rop-ALD-HBD	> 15.64 ± 1.00	< 7.0 ± 1.00	< 7.0 ± 1.00	–
13	Rop-RBM-Rop-Tri-HBD	–	–	–	11.0 (5; 20)
14	RBD	–	–	–	44.2 (20; 320)

Notes. ELISA data are presented as geometric mean titer (GMT) for the group; (log_2_) ± standard geometric deviation; data on virus neutralization are shown as GMT and scatter of titer values for sera; ELISA data for Rop-D2-Rop-Tri-HBD, Rop-D3-Rop-Tri-HBD, Rop-D2-Rop-ALD-HBD, and Rop-D3-Rop-ALD-HBD were obtained previously [7] and are presented for comparison. Dash indicates that the protein was not used in the respective experiment.

**Fig. 3.**
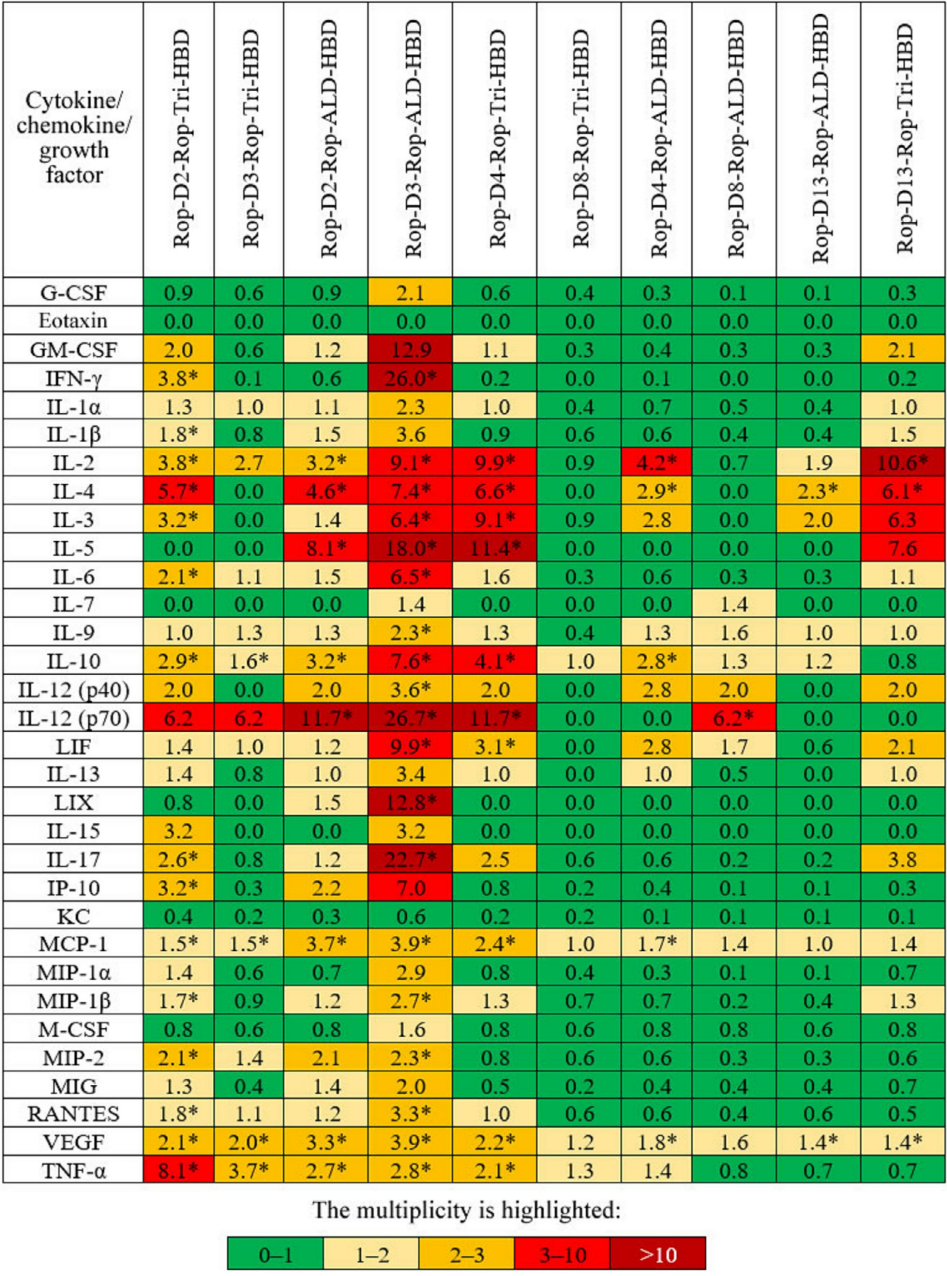
Changes (fold) in the median content of cytokines/chemokines/growth factors in mice immunized with different antigens in comparison with the control group. * Statistically significant difference with the control group (*p* < 0.05) according to the Tukey’s test.

**Cell-mediated immunity.** Activation of cell-mediated immunity was evaluated by measuring the level of cytokines synthesized by splenocytes of immunized mice. Splenocytes (macrophages, dendritic cells, T and B lymphocytes) isolated from the mouse spleen 2 weeks after immunization with antigens mixed with adjuvant 1 were exposed for 24 h to the same antigens, and their response to the stimulation was estimated from the content of 32 regulatory, pro- and anti-inflammatory cytokines, chemokines, and growth factors in the growth medium (Fig. 3). The most pronounced response was observed for Rop-D3-Rop-ALD-HBD; slightly less increase in the level of cytokines was found for Rop-D2-Rop-Tri-HBD, Rop-D3-Rop-Tri-HBD, Rop-D2-Rop-ALD-HBD, Rop-D4-Rop-Tri-HBD, Rop-D4-Rop-ALD-HBD, and Rop-D13-Rop-Tri-HBD.

The maximal response to Rop-D3-Rop-ALD-HBD (Fig. 3) was in agreement with the maximal titer of anti-RBD antibodies demonstrated for this protein (Table 2).

Therefore, the highest cellular response was caused by the hybrid proteins Rop-D2-Rop-Tri-HBD, Rop-D3-Rop-Tri-HBD, Rop-D2-Rop-ALD-HBD, Rop-D3-Rop-ALD-HBD, Rop-D4-Rop-Tri-HBD, Rop-D4-Rop-ALD-HBD, and Rop-D13-Rop-Tri-HBD. A question remained whether the antibodies formed in mice after immunization with these proteins exhibited virus-neutralizing activity.

**Microneutralization reaction.** Virus-neutralizing antibodies in the sera of immunized mice were detected by the microneutralization assay based on TCID50.

In the first microneutralization reaction, we analyzed the sera obtained after the first immunization experiment (three-dose immunization with 2-week intervals, adjuvant 1) and sera obtained previously after immunization with the proteins carrying D2 and D3 epitopes (adjuvant 1) [7]. None of the tested sera neutralized SARS-CoV-2 under assay conditions (see “Materials and Methods” section).

In the second microneutralization reaction, we analyzed the sera obtained in the second immunization experiment (three-dose immunization with 2-week intervals, but with adjuvant 2) using recombinant proteins with the investigated epitopes, previously isolated recombinant proteins with the D2 and D3 epitopes [7], Rop-RBM-Rop-Tri-HBD, and commercial preparation of RBD (Mount Sinai). The results of microneutralization assay are presented in Table 2. Prevention of the CPE of the live virus (hCoV-19/Russia/StPetersburg-3524/2020 EPI_ISL_415710 GISAID) was observed for some sera dilutions after adjuvant modification, which indicated that specific antibodies capable of neutralizing clinical isolate of the virus were formed in response to immunization with the hybrid proteins containing small antigenic determinants of the SARS-CoV-2 S protein. The highest values of the geometric mean titer (GMT) were demonstrated for the sera obtained after immunization with Rop-D13-Rop-Tri-HBD (6.7), Rop-D2-Rop-Tri-HBD (7.4), and Rop-D4-Rop-Tri-HBD (8.2). The GMT values for the sera obtained by immunization with the control preparations of Rop-RBM-Rop-Tri-HBD and recombinant RBD were 11.0 and 44.2, respectively (Table 2).

## DISCUSSION

In this study, we expanded the list of investigated surface epitopes of the SARS-CoV-2 S protein: four epitopes – D4 (a.a. 496-507), D6 (a.a. 144-153), D8 (a.a. 337-346), and D13 (a.a. 414-425) – were added to the previously studied epitopes D2 (a.a. 470-490) and D3 (a.a. 453-494) [7] (Fig. 1).

An important requirement in the epitope selection was that they had to include amino acids interacting with ACE2 and virus-neutralizing antibodies. The binding of RBD with ACE2 is mediated through amino acids residues at the following positions: 417, 446, 449, 453, 455, 456, 475, 486, 487, 489, 493, 496, 498, 500, 502, 504, and 505 [10]. The majority of these residues were in the selected D2, D3, D4, D8, and D13 epitopes. A number of neutralizing antibodies described in the literature bind to the RBD epitopes overlapping with the epitopes analyzed in our studies: P2B-2F6 [11], BD23 [12], COVA2-39 [13], REGN10933 and REGN10987 [14], LY-CoV555 [15].

The N-terminal domain (NTD) of the S1 subunit of S protein is limited to the residues 20-286. Several neutralizing antibodies have been isolated that bind epitopes located in this fragment: 4A8 [16], COV2-2676 and COV2-2489 [17], S2L28, S2M28 and S2X333 [18]. Therefore, designing the constructs with epitopes corresponding to the NTD (D6) also seems promising.

All epitopes except D8 (α-helix) had a loop-like conformation in the S protein structure with the N- and C-termini brought close together. The conformation of the selected epitopes was fixed by inserting them into the turn between the two interacting α-helices of the Rop-like protein from *M. capsulatus*. The trimerizing module (either ALD from *T. maritima* or trimerizing α-helix from the S protein) was attached to the construct at the C-terminus. A fragment of hemagglutinin from *M. tuberculosis* was placed at the C-terminus of the construct with the trimerizing module to facilitate purification of hybrid proteins by heparin-Sepharose affinity chromatography.

It is interesting to note that despite the fact that all produced hybrid proteins caused formation of high-titer IgGs that interacted with the corresponding antigens, only proteins with the D2 and D3 induced antibodies with a titer sufficient for virus inactivation. The antibodies induced by proteins with the D2 and D3 epitopes and Rop-D13-Rop-Tri-HBD also interacted with the RBD synthesized in prokaryotic cells. The same proteins, as well as Rop-D4-Rop-ALD-HBD and Rop-D4-Rop-Tri-HBD, caused a highly pronounced increase in the level of cytokines in the cell-mediated immunity activation assay. The development of innate immune response with potential differentiation of B cells was observed as a result of splenocyte stimulation by Rop-D3-Rop-ALD-HBD (and to a lesser degree, by other proteins). We should also mention the presence of very early signs of development of other components of adaptive immune response, in particular, the signs of differentiation of T cells into Th1 cells (IFN-γ), as well as appearance of cytokines typical for Th2 and Th17 cells (IL-4, IL-5, and IL-17). The presence of stimulatory (IL-1 and IL-12) and suppressor (IL-10) cytokines implies triggering of the full-fledged immune response by the antigens. However, despite the pronounced immune response, the sera obtained after immunization of mice with the compositions containing adjuvant 1 displayed no virus-neutralizing activity in the microneutralization assay.

Selection of optimal adjuvant in the process of vaccine formulation could be of crucial importance for the development of immune response and formation of protective antibodies against certain pathogens [19]. It was found that agonists of the Toll-like receptors (TLRs) could be used as adjuvant components to increase the efficiency of UV-inactivated vaccine against SARS-CoV [20] and vaccine composition based on the recombinant protein containing the ectodomain of the SARS-CoV S protein [6]. However, immunization of mice with the ectodomain mixed with gold nanoparticles (adjuvant) did not generate protective antibodies [6]. Considering the above information, we modified the adjuvant by replacing montanide ISA 201 and retinol palmitate with TLR agonists – LPS from *E. coli* O55:B5 and CpG oligodeoxynucleotides (ODN 1826). In the case of adjuvant 2, DEAE-dextran 500 (which is present in both adjuvants) also facilitated deposition of CpG oligonucleotides at the site of introduction. Immunization of mice with the hybrid proteins using adjuvant 2 resulted in the generation of sera, some of which ensured efficient neutralization of the clinical isolate of SARS-CoV-2 in the microneutralization assay (Table 2). The most pronounced neutralizing activity was found for the sera produced by immunization with the Rop-D13-Rop-Tri-HBD, Rop-D2-Rop-Tri-HBD, and Rop-D4-Rop-Tri-HBD proteins. Interestingly, all hybrid proteins causing formation of neutralizing antibodies had a relatively small S protein trimerization domain, rather than the relatively large ALD domain, thus suggesting that the use of smaller protein carriers that shift the ratio towards the dominance of the protein antigenic fragment, could be more productive. It is possible that this can be due to the fact that the trimerization domain is a part of the S protein.

Hybrid proteins that caused the most pronounced immune response with the formation of neutralizing antibodies contained short loop-like epitopes located within the RBM boundaries (D2 and D4) or in the RBD region preceding RBM (D13). These epitopes overlap significantly with the linear epitopes S406-420, S475-499, and S495-509 reported by Lu et al. [5] (Fig. 1). According to the data presented in [5], all these linear epitopes are virus-neutralizing with respect to the SARS-CoV-2 variant G614 [5]. This is in good agreement with our results on neutralization of the clinical isolate hCoV-19/Russia/StPetersburg-3524/2020 EPI_ISL_415710 GISAID with the same D614G substitution. The highest ability for the induction of neutralizing antibodies (GMT close to that of the protein with the full-size RBM) was found for the protein with the short loop-like D4 epitope.

All epitopes selected in this study contained amino acid residues mutated in the S protein of the Omicron variant of SARS-CoV-2 (B.1.1.529) that has caused the highest wave of COVID-19 in comparison with the previous virus variants [21]. Moreover, most of variable amino acid residues were located in the epitopes with the maximal virus-neutralizing activity (Fig. 1). This is not surprising, because all selected determinants are located on the S protein surface, while the RBD region containing all virus-neutralizing epitopes is directly involved in the interaction with ACE2. High occurrence of variable sites in the region of neutralizing epitopes would allow to rapidly produce modified sequences based on the existing constructs and to correct the neutralizing response in order to target a corresponding coronavirus mutant. Possibly, it would make sense to expand the boundaries of epitopes and to use entire RBM with the maximal number of variable amino acid residues for the development of epitope vaccine. This can be a reasonable approach because RBM contains no glycosylation sites. Recombinant RBM protein is efficiently produced in *E. coli*; it could be highly purified and subjected to correct re-folding with the formation of disulfide bond. It exhibits the highest GMT in the microneutralization assay among all hybrid proteins produced via microbiological synthesis.

It should be mentioned that despite the results of microneutralization assay, which demonstrate the possibility of formation of measurable amounts of neutralizing antibodies after immunization with recombinant proteins carrying small loop-like epitopes of the RBD, the GMT values of the obtained sera were lower in comparison with the GMT value for the full-size RBD synthesized in eukaryotic cells. This defines the need for future studies on the development of epitope vaccines for prevention of coronavirus infection that could include the following directions: (i) search for new promising virus-neutralizing epitopes in SARS-CoV-2 proteins, e.g., based on the experimental data on linear epitopes that exhibit good correlation with the data on conformational epitopes; (ii) combined use of several hybrid proteins for immunization with varying doses of antigens; (iii) enlargement of loop-like epitopes (one of which can be RBM itself); (iv) optimization of epitope multimerization platform.

Considering that selection of adjuvant to facilitating formation of virus-neutralizing antibodies even with very short sequences of the SARS-CoV-2 S protein used as antigens, was one of the most significant results of this work, it would be interesting to characterize the immunogenicity of proteins causing formation of neutralizing antibodies, to evaluate the ability of the corresponding for RBD binding and virus inactivation, and to estimate the effect of immunization with these proteins on the cell-mediated immunity, which might help in predicting the neutralizing activity of proteins based on simpler experiments that do not require live virus. This could become another direction of future research.

In conclusion, we demonstrated high immunogenicity of hybrid recombinant proteins containing small loop-like conformational epitopes of RBD and N-terminal domain of the SARS-CoV-2 S protein. Some of the recombinant proteins induced production of high-titer antibodies capable of binding inactivated SARS-CoV-2 and RBD synthesized in eukaryotic cells. Activation of cell-mediated immunity by immunization with the recombinant proteins was evaluated based on the level of cytokine synthesis by splenocytes from the immunized mice. Modification of the adjuvant, which included introduction of LPS and CpG oligonucleotides (TLR agonists), resulted in the formation of antibodies with the neutralizing activity against live SARS-CoV-2 as demonstrated by the microneutralization assay.

## Abbreviations

ACE2: angiotensin converting enzyme 2
ALD: aldolase
DTT: dithiothreitol
COVID-19: COronaVIrus Disease 2019
HBD: heparin-binding domain
RBD: receptor-binding domain
RBM: receptor-binding motif
SARS-CoV-2: severe acute respiratory syndrome coronavirus 2
Tri: α-helix mediating trimerization of SARS-CoV-2 Spike protein
